# Psychosis associated with acute recreational drug toxicity: a European case series

**DOI:** 10.1186/s12888-016-1002-7

**Published:** 2016-08-18

**Authors:** Odd Martin Vallersnes, Alison M. Dines, David M. Wood, Christopher Yates, Fridtjof Heyerdahl, Knut Erik Hovda, Isabelle Giraudon, Euro-DEN Research Group, Jacek Sein Anand, Alan Blake, Lucie Chevillard, Florian Eyer, Miguel Galicia, Catalina Homar, Gesche Jürgens, E. Liakoni, M. E. Liechti, Gerard Markey, Bruno Mégarbane, Oscar Miro, Adrian Moughty, Niall O’Connor, Raido Paasma, Carsten Boe Pedersen, Kristiina Pöld, Jordi Puiguriguer, Roumen Sedefov, Jochen Stenzel, Wojciech Waldman, W Stephen Waring, Paul I. Dargan

**Affiliations:** 1 Department of General Practice, University of Oslo, Oslo, Norway; 2 Oslo Accident and Emergency Outpatient Clinic, City of Oslo Health Agency, Oslo, Norway; 3 Clinical Toxicology, Guy’s and St Thomas’ NHS Foundation Trust and King’s Health Partners, London, UK; 4 Clinical Toxicology, Faculty of Life Sciences and Medicine, King’s College London, London, UK; 5 Emergency Department and Clinical Toxicology Unit, Hospital Universitari Son Espases, Mallorca, Spain; 6 The Norwegian CBRNe Centre of Medicine, Oslo University Hospital, Oslo, Norway; 7 European Monitoring Centre for Drugs and Drug Addiction (EMCDDA), Lisbon, Portugal

**Keywords:** Psychosis, Psychostimulants, Hallucinogens, Novel psychoactive substances, Amphetamine, Recreational drugs, Acute poisoning, Acute toxicity, Substance use disorders

## Abstract

**Background:**

Psychosis can be associated with acute recreational drug and novel psychoactive substance (NPS) toxicity. However, there is limited data available on how common this is and which drugs are most frequently implicated. We describe a European case series of psychosis associated with acute recreational drug toxicity, and estimate the frequency of psychosis for different recreational drugs.

**Methods:**

The European Drug Emergencies Network (Euro-DEN) collects data on presentations to Emergency Departments (EDs) with acute recreational drug and NPS toxicity at 16 centres in ten countries. Euro-DEN data from October 2013 through September 2014 was retrospectively searched, and cases with psychosis were included. The proportion of cases with psychosis per drug was calculated in the searched Euro-DEN dataset.

**Results:**

Psychosis was present in 348 (6.3 %) of 5529 cases. The median (interquartile range) age was 29 (24-38) years, 276 (79.3 %) were male and 114 (32.8 %) were admitted to psychiatric ward. The drugs most commonly reported were cannabis in 90 (25.9 %) cases, amphetamine in 87 (25.0 %) and cocaine in 56 (16.1 %). More than one drug was taken in 189 (54.3 %) cases. Psychosis was frequent in those ED presentations involving tryptamines (4/7; 57.1 %), methylenedioxypyrovalerone (MDPV) (6/22; 27.3 %), methylphenidate (6/26; 23.1 %), lysergic acid diethylamide (LSD) (18/86; 20.9 %), psilocybe mushrooms (3/16; 18.8 %), synthetic cannabinoid receptor agonists (4/26; 15.4 %) and amphetamine (87/593; 14.7 %), but less common in those involving mephedrone (14/245; 5.7 %), methylenedioxymethamphetamine (MDMA) (20/461; 4.3 %) and methedrone (3/92; 3.3 %). Amphetamine was the most frequent drug associated with psychosis when only one agent was reported, with psychosis occurring in 32.4 % of these presentations.

**Conclusion:**

The frequency of psychosis in acute recreational drug toxicity varies considerably between drugs, but is a major problem in amphetamine poisoning. In rapidly changing drug markets and patterns of use, the Euro-DEN sentinel network contributes to measuring the scale of drug-related harms in Europe beyond other more established indicators.

## Background

Acute psychosis is a serious medical condition associated with significant morbidity [1]. Use of classic sympathomimetic drugs like amphetamine, methamphetamine and cocaine can induce acute psychosis [2–4]. Whilst the prevalence of psychosis in the community has been estimated at about 0.5 % [5, 6], the prevalence of drug induced psychosis among regular drug users has been reported to be in the range of 8-46 % for amphetamine [2] and 7-86 % for cocaine [3]. The wide ranges are probably due to variations in methods of data collection and the populations studied [2, 3]. Recent decades have seen an increased potency of cannabis preparations [7–9], carrying an increased risk of psychosis [10]. Though most drug induced psychoses resolve within a few days, as many as 8-27 % have been reported to persist for more than one month [11–13].

Over the last decade there has been an increase in the availability and use of a multitude of novel psychoactive substances (NPS), with 101 new substances reported to the European Monitoring Centre for Drugs and Drug Addiction (EMCDDA) in 2014 [8]. These NPS encompass a variety of drug classes, including the cathinones, phenethylamines, tryptamines, piperazines and synthetic cannabinoid receptor agonists (SCRA) [14–17]. There have been numerous reports of acute NPS toxicity associated with psychosis, involving several different drugs, among them methylenedioxypyrovalerone (MDPV), mephedrone, alpha-methyltryptamine (AMT), 6-(2-aminopropyl)benzofuran (6-APB), diphenylprolinol (D2PM), and SCRAs [17–28]. However, there is limited data available on how common psychosis is in patients presenting with acute recreational drug or NPS toxicity and which drugs are most frequently implicated.

The European Drug Emergencies Network (Euro-DEN) is a European Commission funded project which collected data on presentations to Emergency Departments (EDs) with acute recreational drug and NPS toxicity in 16 sentinel centres in ten European countries [29]. This has been continued as the Euro-DEN Plus network following completion of the initial project, with the aim of increasing the number of centres and coverage across Europe.

## Methods

The aim of this study was to describe the cases of psychosis associated with acute recreational drug and NPS toxicity reported in the first year of the Euro-DEN project and to estimate the psychosis rate in acute toxicity presentations with different recreational drugs and NPS.

We have previously described the Euro-DEN methodology in detail [29]. The participating centres were mainly hospital emergency departments, some were specialist toxicology units, and one was a primary care emergency outpatient clinic. For the remainder of this article we will use the term recreational drugs to encompass both classical recreational drugs and NPS.

### Inclusion

Patients presenting to a participating Euro-DEN centre with symptoms and/or signs consistent with acute recreational drug toxicity and/or directly related to recreational drug use were included in Euro-DEN; presentations with isolated ethanol intoxication were excluded. Recreational drugs were defined as any psychoactive compound taken for recreational rather than for medical or work purposes or deliberate self-harm (this included classical recreational drugs, NPS and prescription medicines); the drug(s) involved in the presentation were based on the patient’s self report and/or the clinical assessment of the treating physician. For each included case we collected a minimum data set of pre-defined demographic, clinical and outcome variables from information in the patients’ medical records. For this study, Euro-DEN data from October 2013 through September 2014 were retrospectively searched, and all presentations with psychosis as a clinical feature were included.

Psychosis was one of the pre-defined clinical features collected in the minimum data set. Psychosis was defined as any episode of delusions, transient or persistent, accompanied by confusion, hallucinations and lack of insight, based on the assessment of the treating clinician as documented in the patient’s notes.

### Data extraction

The data extracted for this study were: age and gender, drugs taken, clinical observations at presentation (temperature, heart rate, blood pressure, respiratory rate, conscious level, blood glucose), clinical features (psychosis, hallucinations, agitation, anxiety, hyperthermia), treatment and outcome (disposition from the ED, length of hospital stay and death in hospital).

### Outcome measures

We calculated the proportion of presentations with psychosis for each drug amongst all presentations in the Euro-DEN dataset where the drug was reported, whether as a sole drug or in combination with others. We also calculated the odds ratio (OR) for presenting with psychosis for each drug.

### Ethics

The study was done in accordance with the Helsinki declaration. Each centre obtained appropriate ethical approval to collect the data from their institution. Written consent from the patients was not necessary, as no data other than that collected as part of the routine clinical examination was being used for the project.

### Statistics

Pearson’s chi-square test or Fisher’s exact test (for expected cell values five or less) were used to compare frequencies. T-test or Mann-Whitney U-test were used in comparisons of continuous variables. Logistic regression analyses were used to estimate the association between psychosis and different recreational drugs. For each drug the patients presenting without psychosis were defined as the reference group (OR = 1). In the multivariate analyses the odds ratios for psychosis being a clinical feature associated with a drug were adjusted for other drugs reported (Table 3). Analyses were done in IBM SPSS version 21 (IBM Corp.) and in an online calculator from EpiTools epidemiological calculators (http://epitools.ausvet.com.au) [30].

## Results

Over the 12 month period there were 5529 cases with acute recreational drug toxicity reported to the Euro-DEN project from the participating centres, and psychosis was recorded as a clinical feature in 348 (6.3 %). Table 1 lists the number of presentations with psychosis at each of the participating centres; the proportion of presentations with psychosis varied from 3.0 to 16.3 %.

**Table 1:** Presentations with psychosis associated with acute recreational drug toxicity, per participating Euro-DEN centre

Centre, Country	Total presentations n	Presentations with psychosis n (%)
Barcelona, Spain	199	22 (11.1)
Basel, Switzerland	216	12 (5.6)
Copenhagen, Denmark	183	6 (3.3)
Drogheda, Ireland	36	4 (11.1)
Dublin, Ireland	526	19 (3.6)
Gdansk, Poland	144	17 (11.8)
London KCH, UK	422	13 (3.1)
London STH, UK	956	29 (3.0)
Mallorca, Spain	181	12 (6.6)
Munich, Germany	214	22 (10.3)
Oslo OAEOC, Norway	1478	113 (7.6)
Oslo OUH, Norway	199	9 (4.5)
Paris, France	454	42 (9.3)
Pärnu, Estonia	15	1 (6.7)
Tallinn, Estonia	104	17 (16.3)
York, UK	202	10 (5.0)
Total	5529	348 (6.3)

KCH: King’s College Hospital NHS Foundation Trust

OAEOC: Oslo Accident and Emergency Outpatient Clinic

OUH: Oslo University Hospital

STH: St Thomas’ Hospital (Guy’s and St Thomas’ NHS Foundation Trust)

UK: United Kingdom of Great Britain and Northern Ireland

Amongst the patients presenting with psychosis, the median (interquartile range (IQR)) age was 29 (24-38) years, and 276 (79.3 %) were male (Table 2). In addition to psychosis, 63.2 % had agitation, and 43.7 % had hallucinations (Table 2). The drugs most commonly reported in the presentations with psychosis were cannabis in 90 (25.9 % of the psychosis presentations), amphetamine in 87 (25.0 %) and cocaine in 56 (16.1 %). More than one drug was taken in 189 (54.3 %) of the presentations with psychosis, including 105 (30.2 %) cases in which ethanol was co-ingested.

**Table 2:** Presentations with psychosis – demographics, clinical data and disposition from the ED

	Presentations with psychosis			Presentations without psychosis			
	n (%)	median (IQR)	mean (SD)	n (%)	median (IQR)	mean (SD)	*p*-value
Age							
Total		29 years (24-38)			31 years (24-39)		0.077^b^
Males	276 (79.3)^a^	29 years (24-38)		3892 (75.1)^a^	32 years (25-39)		0.041^b^
Females	72 (20.7)^a^	26.5 years (22-39)		1289 (24.9)^a^	28 years (22-37)		0.675^b^
Observations at presentation							
Respiratory rate			18 /min (5)			16 /min (5)	<0.001
Heart rate			100 /min (22)			91 /min (24)	< 0.001
Systolic blood pressure			131 mmHg (20)			125 mmHg (21)	< 0.001
Diastolic blood pressure			80 mmHg (16)			77 mmHg (16)	0.004
Temperature			36.7 °C (0.8)			36.3 °C (0.9)	< 0.001
Blood glucose		5.7 mmol/L (5.1-6.9)			5.6 mmol/L (4.9-6.7)		0.079
*Conscious level*							
Alert (GCS 15)	203 (58.3)			2564 (49.5)			0.002
Drowsy (GCS 8-14)	122 (35.1)			2013 (38.9)			0.002
Comatose (GCS ≤ 7)	9 (2.6)			448 (8.6)			< 0.001
Not recorded	14 (4.0)			156 (3.0)			0.369
Clinical features							
Hallucinations	152 (43.7)			232 (4.5)			< 0.001
Agitation/aggression	220 (63.2)			1247 (24.1)			< 0.001
Anxiety	129 (37.1)			911 (17.6)			< 0.001
Hyperthermia	7 (2.0)			67 (1.3)			0.375
Disposition from ED							
Medically discharged	132 (37.9)			3016 (58.2)			< 0.001
Self discharge	50 (14.4)			906 (17.5)			0.157
Admitted critical care	14 (4.0)			318 (6.1)			0.140
Admitted psychiatric ward	114 (32.8)			170 (3.3)			< 0.001
Admitted other	38 (10.9)			739 (14.3)			0.097
Death	-			16 (0.3)			0.601
Unknown	-			16 (0.3)			0.601
Length of stay in hospital		5.1 hours (2.3-13.6)			4.6 hours (2.5-9.7)		0.176
Total	348 (100)			5181 (100)			

^a^No significant difference in gender proportions between presentations with and without psychosis (*p* = 0.091)

^b^ *p*-value for comparison of age

ED: emergency department; GCS: Glasgow Coma Scale; IQR: interquartile range; SD: standard deviation

The strongest associations with psychosis were found for tryptamines (psychosis in 57.1 % of presentations; adjusted OR 12.4), MDPV (27.3 %; adjusted OR 6.5), methylphenidate (23.1 %; adjusted OR 3.9), lysergic acid diethylamide (LSD) (20.9 %; adjusted OR 3.1), amphetamine (14.7 %; adjusted OR 3.0), and methamphetamine (11.3 %; adjusted OR 2.3) (Table 3).

**Table 3:** Drugs associated with psychosis – logistic regression analysis

	All presentations where the drug was reported		Crude			Adjusted			Presentations where the drug was the single agent reported	
Drug	Total n (%)	With psychosis n (%)	Odds ratio	95 % CI	*p*-value	Odds ratio	95 % CI	*p*-value	Total n (%)	With psychosis n (%)
Tryptamines	7 (0.1)	4 (57.1)	20.1	4.5 – 90.0	< 0.001	**12.4**	2.3 – 65.2	0.003	2 (0.1)	2 (100)
MDPV	22 (0.4)	6 (27.3)	5.7	2.2 – 14.6	< 0.001	**6.5**	2.2 – 19.2	0.001	2 (0.1)	2 (100)
Methylphenidate	26 (0.5)	6 (23.1)	4.5	1.8 – 11.3	0.001	**3.9**	1.5 – 10.2	0.006	5 (0.2)	2 (40.0)
LSD	86 (1.6)	18 (20.9)	4.1	2.4 – 7.0	< 0.001	**3.1**	1.8 – 5.5	< 0.001	27 (1.3)	7 (25.9)
Amphetamine	593 (10.7)	87 (14.7)	3.1	2.4 – 4.0	< 0.001	**3.0**	2.3 – 4.0	< 0.001	136 (6.7)	44 (32.4)
Methamphetamine	186 (3.4)	21 (11.3)	2.0	1.2 – 3.1	0.005	**2.3**	1.4 – 3.8	0.001	44 (2.2)	8 (18.2)
Psilocybe mushrooms^a^	16 (0.3)	3 (18.8)	3.5	0.98 – 12.2	0.054	2.2	0.60 – 8.0	0.24	6 (0.3)	1 (16.7)
SCRA	26 (0.5)	4 (15.4)	2.7	0.93 – 8.0	0.066	1.8	0.61 – 5.4	0.29	17 (0.8)	3 (17.6)
Cannabis	904 (16.4)	90 (10.0)	1.9	1.5 – 2.4	< 0.001	**1.5**	1.2 – 2.0	0.002	216 (10.6)	31 (14.4)
Z-drugs	106 (1.9)	8 (7.5)	1.2	0.59 – 2.5	0.59	1.4	0.68 – 3.1	0.34	16 (0.8)	-
Fentanyl	47 (0.9)	4 (8.5)	1.4	0.50 – 3.9	0.53	1.3	0.44 – 3.9	0.64	10 (0.5)	2 (20.0)
Tramadol	34 (0.6)	3 (8.8)	1.4	0.44 – 4.7	0.55	1.3	0.39 – 4.7	0.64	5 (0.2)	1 (20.0)
Crack	136 (2.5)	6 (4.4)	0.68	0.30 – 1.6	0.36	1.1	0.45 – 2.5	0.89	24 (1.2)	3 (12.5)
Cocaine	957 (17.3)	56 (5.9)	0.91	0.68 – 1.2	0.54	0.93	0.68 – 1.3	0.65	164 (8.1)	11 (6.7)
Pregabalin	79 (1.4)	4 (5.1)	0.79	0.29 – 2.2	0.65	0.92	0.31 – 2.8	0.88	3 (0.1)	-
Mephedrone	245 (4.4)	14 (5.7)	0.90	0.52 – 1.6	0.70	0.90	0.50 – 1.6	0.72	72 (3.5)	4 (5.6)
Buprenorphine	89 (1.6)	6 (6.7)	1.1	0.47 – 2.5	0.86	0.90	0.36 – 2.2	0.82	16 (0.8)	1 (6.3)
Ketamine	128 (2.3)	5 (3.9)	0.60	0.24 – 1.5	0.27	0.59	0.23 – 1.5	0.26	12 (0.6)	-
MDMA	461 (8.3)	20 (4.3)	0.66	0.41 – 1.0	0.073	**0.58**	0.36 – 0.95	0.030	59 (2.9)	-
Benzodiazepines	1011 (18.3)	34 (3.4)	0.47	0.33 – 0.67	< 0.001	**0.53**	0.36 – 0.78	0.001	79 (3.9)	2 (2.5)
Methedrone	92 (1.7)	3 (3.3)	0.50	0.16 – 1.6	0.24	0.49	0.15 – 1.6	0.24	21 (1.0)	1 (4.8)
GHB/GBL	710 (12.8)	25 (3.5)	0.51	0.34 – 0.77	0.001	**0.38**	0.25 – 0.59	< 0.001	215 (10.6)	4 (1.9)
Methadone	248 (4.5)	6 (2.4)	0.36	0.16 – 0.81	0.014	**0.34**	0.14 – 0.80	0.014	38 (1.9)	1 (2.6)
Heroin	1344 (24.3)	21 (1.6)	0.19	0.12 – 0.29	< 0.001	**0.18**	0.11 – 0.28	< 0.001	564 (27.8)	8 (1.4)
Other cathinones	20 (0.4)	4 (20.0)	3.8	1.2 – 11.3	0.019	3.0	0.97 – 9.4	0.057	10 (0.5)	1 (10.0)
Other amphetamine derivatives	27 (0.5)	4 (14.8)	2.6	0.90 – 7.6	0.078	1.8	0.57 – 5.5	0.33	4 (0.2)	2 (50.0)
Other stimulants	36 (0.7)	5 (13.9)	2.4	0.94 – 6.4	0.068	2.0	0.75 – 5.3	0.17	8 (0.4)	3 (37.5)
Other hallucinogens	55 (1.0)	8 (14.5)	2.6	1.2 – 5.5	0.015	**2.3**	1.0 – 5.1	0.040	20 (1.0)	3 (15.0)
Ethanol^b^	2145 (38.8)	105 (4.9)	0.66	0.53 – 0.84	< 0.001	**0.54**	0.42 – 0.69	< 0.001	-	-
Other opioids	204 (3.7)	5 (2.5)	0.36	0.15 – 0.89	0.031	**0.30**	0.12 – 0.75	0.010	51 (2.5)	-
Total	5529 (100)^c^	348 (6.3)							2030 (100)	159 (7.8)

Adjusted odds ratios with *p* < 0.05 are given in bold types

CI: confidence interval

GBL: gamma-butyrolactone

GHB: gamma-hydroxybutyrate

LSD: lysergic acid diethylamide

MDMA: methylenedioxymethamphetamine

MDPV: methylenedioxypyrovalerone

NPS: novel psychoactive substances

SCRA: Synthetic cannabinoid receptor agonists

^a^Includes three presentations reporting psilocybin

^b^Ethanol only included as co-ingestion

^c^Percentages do not add up to 100 as in several cases more than one drug was reported

In the 159 single agent cases, the largest proportions of presentations with psychosis were found for tryptamines (2/2; 100 %), MDPV (2/2;100 %), methylphenidate (2/5; 40.0 %), amphetamine (44/136; 32.4 %), and LSD (7/27; 25.9 %) (Table 3). For benzodiazepines the proportion of presentations with psychosis was small (3.4 %), and nearly all (94.1 %) benzodiazepine cases with psychosis were in combination with other drugs.

Treatment was required in 192 (55.2 %) of the presentations with psychosis, including sedation in 154 (44.3 %). The most common sedatives/antipsychotics given were benzodiazepines in 127 (36.5 %) cases, olanzapine in 27 (7.8 %), haloperidol in 26 (7.5 %) and propofol in 14 (4.0 %). The median length of stay in hospital was 5.1 hours (IQR 2.3-13.6). In 132 (37.9 %) cases the patient was medically discharged from the ED, a significantly lower proportion than the 58.2 % among those without psychosis (*p* < 0.001) (Table 2). In 114 (32.8 %) cases the patient was admitted to a psychiatric ward, compared to 3.3 % among those without psychosis (*p* < 0.001). There was no difference in the proportions admitted to critical care.

## Discussion

Psychosis was a clinical feature in 6.3 % of this European case series of 5529 presentations to an ED with acute recreational drug toxicity. The association with psychosis varied considerably between drugs. Not surprisingly, the associations were strongest for hallucinogens and some central stimulants. Overall, cannabis, amphetamine and cocaine were the drugs most commonly involved in presentations with psychosis. Amphetamine was the drug most frequently involved in single agent presentations, with 32.4 % single agent amphetamine cases presenting with psychosis. Amongst the NPS, psychosis was rare in presentations involving mephedrone and methedrone, but occurred more frequently with tryptamines, MDPV, methylphenidate and the SCRAs.

Among the patients presenting with recreational drug toxicity and psychosis, 63.2 % were agitated and/or aggressive (compared to 24.1 % of those without psychosis), 43.7 % had hallucinations (compared to 4.5 %), and 32.8 % were admitted to a psychiatric ward (compared to 3.3 %), indicating that this is a group of patients with substantial resource implications. Furthermore, acute psychosis induced by substance use is, until it has resolved, difficult to distinguish clinically from schizophrenic psychosis [31, 32]. Some authors have questioned whether they are separate entities at all [2, 33–35]. Longer duration of untreated psychosis is associated with poorer outcome, though a causal relationship has not been established [36]. However, there is evidence suggesting that early intervention, reducing the duration of untreated psychosis, could be helpful [37–39].

Cannabis, amphetamine and cocaine were the drugs most commonly involved in presentations with psychosis in our study. This is to be expected as they are commonly used in Europe [8] and carry the risk of inducing acute psychosis [2, 3, 40]. We found amphetamine more frequently associated with psychosis than cannabis and cocaine, both as a single agent and in the logistic regression analysis, confirming that psychosis is a major feature of acute amphetamine toxicity. Cannabis, but not cocaine, was significantly associated with psychosis in the logistic regression analysis.

Among the NPS, tryptamines, MDPV and SCRAs were the drugs most frequently involved in presentations with psychosis. In our study, 57.1 % of patients with tryptamine poisoning presented with psychosis, a larger proportion than the 23.6 % reported among 55 enquiries concerning AMT in a poison centre case series in the UK [27]. This substantiates previous reports noting psychosis as a prominent clinical feature of acute tryptamine toxicity [41]. In a study of 23 patients with acute MDPV toxicity seen by medical toxicologists in the US, psychosis was reported in 8.7 % [28]. This is less than the 27.3 % in our study, and our findings are more in keeping with a US poison centre study of 236 cases of MDPV toxicity, where 36 % had paranoia, 34 % confusion and 40 % hallucinations [18]. In our study, 15.4 % of those presenting with acute SCRA toxicity had psychosis – this is comparable to the 9-11 % reporting hallucinations and delusion to US poison centres in two case series of 1353 and 418 acute SCRA exposures [23, 24]. However, a German case series of 29 ED presentations with laboratory confirmed acute SCRA toxicity reported only one case of acute psychosis though 38 % of the patients had hallucinations or changes of perception [42]. For mephedrone, 5.7 % of patients in our study presented with psychosis, slightly less than the 8 % reported among 488 enquiries to UK poison centres [27] and the 9 % with paranoia and hallucinations among 57 presentations to the ED in a Scottish case series [43]. No psychotic features were reported among seven cases with laboratory confirmed mephedrone toxicity in a London ED [44].

Psychosis has previously been reported as a feature of acute methylphenidate toxicity as well as a potential side effect of methylphenidate treatment [45]. In our study, 23.1 % of presentations involving methylphenidate had psychosis.

Tramadol and fentanyl differed from the other opioids, with a larger proportion of presentations with psychosis. Though only seven cases with psychosis were seen involving these drugs, two were single agent cases with fentanyl and one with tramadol. We have only found one previous report of psychosis as a clinical feature of tramadol toxicity [46], and none involving fentanyl.

In addition to the risk of inducing acute psychosis, regular use of several recreational drugs, especially amphetamine, methamphetamine and cannabis, has been shown to be associated with later development of chronic psychosis or schizophrenia [2, 11, 34, 47]. Japanese studies have reported schizophrenia-like psychosis in methamphetamine users persisting for months to years after discontinuation of methamphetamine use [11, 33]. A large cohort study in California found an increased risk of later being diagnosed with schizophrenia in previously non-psychotic hospitalised users of methamphetamine or cannabis [34]. A meta-analysis of seven studies found cannabis use to be an independent risk factor for later development of chronic psychosis [47]. The risk was higher the more cannabis was used, and the earlier in life the start of the exposure [47]. It also seems that patients with previous substance use associated psychosis are at greater risk of subsequent psychotic episodes [2, 33].

### Limitations

Data on previous psychiatric diagnoses were not collected. Therefore, we are not able to determine whether the psychosis seen at the time of the acute recreational drug toxicity presentation was an acute psychosis, the patient’s regular psychotic state or an exacerbation of a chronic psychosis. Furthermore, we did not collect information on the frequency or duration of drug use. It is possible that large proportions of presentations with psychosis reflect drugs of choice for patients with chronic psychosis [48]. We do not have any follow-up data for the patients in our study. However, the short length of stay and high proportion of patients medically discharged from the ED suggests that in a substantial proportion the psychosis was of limited duration.

The diagnosis of psychosis was made by the treating ED clinician, and not necessarily by a psychiatrist. Consequently, there is a potential for misdiagnosis; although this represents routine clinical practice and assessment/management of patients in European EDs with acute recreational drug toxicity. There may also be inconsistencies in the diagnosis of psychosis across countries. This may possibly contribute to the variation of psychosis rates between centres seen in our material. The diagnosis of psychosis was mainly based on the assessment of the clinician treating the patient. Therefore, some inter-rater variability must be expected. On the other hand, our results are based on decisions made in real clinical situations, strengthening their generalizability.

Drug(s) involved in the presentations were based on patient self-report. Patients may not report all drugs ingested, and may not know the actual psychoactive ingredient in the substance(s) taken. There is the potential that this will lead to some misclassification. However, this reflects the actual clinical situation in most settings treating acute poisoning, as well as current best practice in toxicology, in which patients are managed based on their clinical presentation.

The proportion of presentations with psychosis is not a measure of a drug’s potential to induce psychosis. Rather, it is a measure of to what extent psychosis is part of the problems associated with the drug’s acute toxicity. For many drugs the numbers in our study are small, and for these drugs the results should be interpreted with caution.

## Conclusions

There is considerable variation between drugs in how commonly psychosis is a feature of acute recreational drug toxicity. Psychosis was seen in a large proportion of presentations with acute amphetamine toxicity. Amongst the NPS, although numbers were small, psychosis was rare in mephedrone and methedrone poisoning, but occurred frequently with tryptamines, MDPV and the synthetic cannabinoid receptor agonists; psychosis was also common in presentations involving methylphenidate.

In rapidly changing drug markets, the Euro-DEN sentinel network contributes to measuring the scale of drug-related harms in Europe beyond other more established European indicators [49–51]. Further study is required in larger cohorts to understand the associations found and consider other factors such as the importance of underlying psychiatric co-morbidity. However, in this series, psychosis was seen in a significant minority of presentations and this has important implications for prevention, follow-up and referral for further treatment, and represents an important burden on health services.

## Abbreviations

6-APB: 6-(2-aminopropyl)benzofuran
AMT: Alpha-methyltryptamine
D2PM: Diphenylprolinol
ED: Emergency department
EMCDDA: The European monitoring centre for drugs and drug addiction
Euro-DEN: The European drug emergencies network
IQR: Interquartile range
LSD: Lysergic acid diethylamide
MDMA: Methylenedioxymethamphetamine
MDPV: Methylenedioxypyrovalerone
NPS: Novel psychoactive substances
OR: Odds ratio
SCRA: Synthetic cannabinoid receptor agonists
