# Erratum to: Psychosis associated with acute recreational drug toxicity: a European case series

**DOI:** 10.1186/s12888-016-1066-4

**Published:** 2016-11-16

**Authors:** Odd Martin Vallersnes, Alison M. Dines, David M. Wood, Christopher Yates, Fridtjof Heyerdahl, Knut Erik Hovda, Isabelle Giraudon, Euro-DEN Research Group, Paul I. Dargan

**Affiliations:** 1 Department of General Practice, University of Oslo, Oslo, Norway; 2 Oslo Accident and Emergency Outpatient Clinic, City of Oslo Health Agency, Oslo, Norway; 3 Clinical Toxicology, Guy’s and St Thomas’ NHS Foundation Trust and King’s Health Partners, London, UK; 4 Clinical Toxicology, Faculty of Life Sciences and Medicine, King’s College London, London, UK; 5 Emergency Department and Clinical Toxicology Unit, Hospital Universitari Son Espases, Mallorca, Spain; 6 The Norwegian CBRNe Centre of Medicine, Oslo University Hospital, Oslo, Norway; 7 European Monitoring Centre for Drugs and Drug Addiction (EMCDDA), Lisbon, Portugal

## Erratum

Unfortunately, after publication of this article [1], it was noticed that the author Paul I. Dargan was missing from the author list in the PDF version of the article. The corrected author list can be seen above and the original article has been updated to correct this error.
